# Socioeconomic Status, Lifestyle, and DNA Methylation Age Among Racially and Ethnically Diverse Adults: NIMHD Social Epigenomics Program

**DOI:** 10.1001/jamanetworkopen.2024.21889

**Published:** 2024-07-29

**Authors:** Alika K. Maunakea, Krit Phankitnirundorn, Rafael Peres, Christian Dye, Ruben Juarez, Catherine Walsh, Connor Slavens, S. Lani Park, Lynne R. Wilkens, Loïc Le Marchand

**Affiliations:** 1 Department of Anatomy, Biochemistry, and Physiology, University of Hawaii at Manoa, John A. Burns School of Medicine, Honolulu; 2 Department of Environmental Health Sciences, Columbia University Mailman School of Public Health, New York, New York; 3 Department of Economics, University of Hawaii at Manoa, Honolulu; 4 Epidemiology Program, University of Hawaii Cancer Center, Honolulu

## Abstract

**Importance:**

Variation in DNA methylation at specific loci estimates biological age, which is associated with morbidity, mortality, and social experiences. Aging estimates known as epigenetic clocks, including the Dunedin Pace of Aging Calculated From the Epigenome (DunedinPACE), were trained on data predominately from individuals of European ancestry; however, limited research has explored DunedinPACE in underrepresented populations experiencing health disparities.

**Objective:**

To investigate associations of neighborhood and individual sociobehavioral factors with biological aging in a racially and ethnically diverse population.

**Design, Setting, and Participants:**

This cohort study, part of the Multiethnic Cohort study conducted from May 1993 to September 1996 to examine racial and ethnic disparities in chronic diseases, integrated biospecimen and self-reported data collected between April 2004 and November 2005 from healthy Hawaii residents aged 45 to 76 years. These participants self-identified as of Japanese American, Native Hawaiian, or White racial and ethnic background. Data were analyzed from January 2022 to May 2024.

**Main Outcomes and Measures:**

DNA methylation data were generated from monocytes enriched from cryopreserved lymphocytes and used to derive DunedinPACE scores from November 2017 to June 2021. Neighborhood social economic status (NSES) was estimated from 1990 US Census Bureau data to include factors such as educational level, occupation, and income. Individual-level factors analyzed included educational level, body mass index (BMI), physical activity (PA), and diet quality measured by the Healthy Eating Index (HEI). Linear regression analysis of DunedinPACE scores was used to examine their associations with NSES and sociobehavioral variables.

**Results:**

A total of 376 participants were included (113 [30.1%] Japanese American, 144 [38.3%] Native Hawaiian, and 119 [31.6%] White; 189 [50.3%] were female). Mean (SE) age was 57.81 (0.38) years. Overall, mean (SE) DunedinPACE scores were significantly higher among females than among males (1.28 [0.01] vs 1.25 [0.01]; *P* = .005); correlated negatively with NSES (*R* = −0.09; *P* = .08), HEI (*R* = −0.11; *P* = .03), and educational attainment (*R* = −0.15; *P* = .003) and positively with BMI (*R* = 0.31; *P* < .001); and varied by race and ethnicity. Native Hawaiian participants exhibited a higher mean (SE) DunedinPACE score (1.31 [0.01]) compared with Japanese American (1.25 [0.01]; *P* < .001) or White (1.22 [0.01]; *P* < .001) participants. Controlling for age, sex, HEI, BMI, and NSES, linear regression analyses revealed a negative association between educational level and DunedinPACE score among Japanese American (β, −0.005 [95% CI, −0.013 to 0.002]; *P* = .03) and Native Hawaiian (β, −0.003 [95% CI, −0.011 to 0.005]; *P* = .08) participants, yet this association was positive among White participants (β, 0.007; 95% CI, −0.001 to 0.015; *P* = .09). Moderate to vigorous PA was associated with lower DunedinPACE scores only among Native Hawaiian participants (β, −0.006; 95% CI, −0.011 to −0.001; *P* = .005), independent of NSES.

**Conclusions and Relevance:**

In this study of a racially and ethnically diverse sample of 376 adults, low NSES was associated with a higher rate of biological aging measured by DunedinPACE score, yet individual-level factors such as educational level and physical activity affected this association, which varied by race and ethnicity. These findings support sociobehavioral interventions in addressing health inequities.

## Key Points

**Question:**

Is the rate of biological aging associated with racial and ethnic differences in social determinants of health?

**Findings:**

In a cohort study among 376 Japanese American, Native Hawaiian, and White adults, low neighborhood socioeconomic status (NSES) was associated with a higher rate of biological aging measured by the DunedinPACE epigenetic clock. Accounting for NSES, race and ethnicity was a significant factor in correlations between diet, educational level, and moderate to vigorous physical activity and DunedinPACE scores.

**Meaning:**

The findings suggest that individual sociobehavioral factors might mitigate negative associations between NSES and biological aging, with differences by race and ethnicity.

## Teaser

This cohort study explores whether the rate of biological aging estimated by an epigenetic clock is associated with social determinants of health in a racially and ethnically diverse population.

## Introduction

Social epigenomics aims to understand the complex interactions of social determinants of health (SDOHs) and behaviors with epigenetic processes that influence health and disease.^1,2,3^ This emerging field posits that racism or social disenfranchisement (proxied via race and ethnicity) shapes personal experiences that affect gene function over the lifespan through epigenetic mechanisms, including small noncoding RNAs, histone modifications, and DNA methylation. Some of these epigenetic modifications underlie biological aging.^4,5,6^ Given technological advances to examine epigenetic variability in population-based studies, reports abound documenting discordance between chronological and biological age, with the latter having a stronger correlation with health.^7^ However, how such differences relate to racial and ethnic health disparities, which remain pervasive in the US, has not been fully elucidated.

Biological aging, which has been associated with morbidity, mortality, health behaviors, and social experiences,^7,8,9,10^ can be estimated from lymphocyte DNA methylation levels at specific genomic loci. Known as epigenetic clocks, these estimates were developed through machine learning on microarray-based DNA methylation data. First-generation Horvath^7,11^ and Hannum^12^ clocks accurately predicted age by cross-sectionally analyzing specific DNA methylation loci across various ages,^12,13^ while second-generation clocks, such as PhenoAge, improved predictions related to biological aging and disease by additionally being trained on biomarker data.^14^ The latest, third-generation clock, Dunedin Pace of Aging Calculated From the Epigenome (DunedinPACE), uniquely measures the rate of aging, having been trained on longitudinal change in biomarkers with DNA methylation data.^15,16^ DunedinPACE estimates of age acceleration, in which biological age advances faster than chronological age, have recently been validated among individuals experiencing swifter declines in physical and cognitive functions.^15,16,17^ However, to our knowledge, this newer clock has yet to be fully examined in racially and ethnically diverse populations typically underrepresented in biomedical research, including those that experience significant environmental, social, and economic inequities that may affect biological aging and underlie health disparities.

Often associated with racial and ethnic disparities in mortality rates, neighborhood socioeconomic status (NSES) incorporates SDOH factors, including educational level, occupation, and income.^18,19,20,21,22,23^ In Hawaii, Native Hawaiian residents have higher rates of diabetes,^24,25^ obesity,^26^ and cardiovascular disease^27,28^ than White residents; those living in low NSES areas have the highest mortality rates, particularly from heart disease.^18^ Meanwhile, Japanese American residents in Hawaii have higher rates of diabetes than White residents^29^ and have the lowest overall mortality in high NSES areas^18^; those in low NSES areas are more likely to have obesity and diabetes than those in higher NSES areas.^30^ Importantly, these cardiometabolic health disparities appear at a significantly younger age among Japanese American and Native Hawaiian residents than among White residents and are associated with adverse sociobehavioral factors,^31^ implicating differences in the rate of biological aging. Prior research has reported associations of SDOHs with biological aging as measured by older-generation clocks,^14,32^ while recent studies have only begun to explore racial and ethnic differences with the new-generation DunedinPACE clock, showing poverty-associated age acceleration in African American populations.^33,34^ In this study, we examined the association of NSES and sociobehavioral factors with biological aging measured by DunedinPACE, the only epigenetic clock to estimate the rate of aging, in a cross-sectional analysis of Hawaii’s multiethnic and understudied population.

## Methods

### Study Population

Described in detail elsewhere,^35^ the Multiethnic Cohort (MEC) study was established from May 1993 to September 1996 to understand the association of race and ethnicity with cancer and chronic disease rates. This cohort study used a cross-sectional analysis of data from included healthy Hawaii residents selected to prospectively examine diabetes development who self-reported as Japanese American, Native Hawaiian, or White; were nonsmokers; and did not have diabetes at blood sample collection between April 2004 and November 2005. Written informed consent was obtained from participants, and this study received approval from the University of Hawaii institutional review board. This study was reported following the Strengthening the Reporting of Observational Studies in Epidemiology (STROBE) reporting guideline.

### Data Collection

At cohort entry (1993-1996), participant data were collected using a detailed 26-page mailed questionnaire on various topics, including demographics, behaviors such as physical activity (PA), and educational level ranging from sixth grade to postgraduate education. Race and ethnicity were self-reported using a standardized survey instrument, and diet quality was measured using the Healthy Eating Index (HEI) 2010, derived from a self-administered food frequency questionnaire.^36^ The HEI score is a measure for adherence to the Dietary Guidelines for Americans; scores range from 0 to 100, with higher scores indicating better alignment with key dietary recommendations that support health. Where indicated, stratification into high and low HEI diet quality groups was based on previous studies.^37^ At blood sample collection (2004-2005), body mass index (BMI; calculated as weight in kilograms divided by height in meters squared) was determined from self-reported height and weight and separated into categories as defined elsewhere^38,39^; participant age was recorded at that time and used in analyses. Moderate or vigorous PA was assessed using categories of high (>16 h/wk) and low (≤16 h/wk) groups as in another study.^40^ The US census data from 1990 (the census closest to evaluation of exposures at baseline and used in subsequent data analysis) were linked to participants’ addresses and analyzed with principal component analysis to create an NSES index comprising educational level, occupation, employment status, household income, poverty, rent, and house value data, as detailed elsewhere.^30^ The NSES index was categorized into quintiles for all Hawaii MEC participants and further defined as low for quintiles 1 to 3 and high for quintiles 4 to 5. Molecular and cell phenotyping data from cryopreserved lymphocytes were generated from November 2017 to June 2021, with data analysis taking place from January 2022 to May 2024.

### Monocyte Isolation and Validation by Flow Cytometry

From each participant, 10 mL of blood was obtained by venipuncture into an acid citrate dextrose tube. Peripheral blood mononuclear cells were isolated using an ACCUSPIN tube (Sigma-Aldrich), cryopreserved, and stored in liquid-phase nitrogen until analysis. The samples were later thawed and used to isolate monocytes using the EasySep Negative Selection, Human Monocyte Enrichment Kit without CD16 depletion and the EasyEights EasySep magnet cell separator (STEMCELL Technologies).^41^ Monocyte purity was verified from 50 000 cell aliquots per sample using flow cytometry as described previously.^42,43^ An enrichment success threshold was set at more than 65% monocytes to limit DNA methylation variability caused by cell-type heterogeneity.^43^

### DNA Methylation Quantification

Nucleic acids from enriched monocytes were extracted using the AllPrep DNA/RNA Mini Kit (Qiagen). DNA samples were bisulfite-converted and hybridized to the Infinium MethylationEPIC BeadChip microarray (Illumina, Inc) as previously described.^42^ Data processing involved R, version 4.1.2 (R Project for Statistical Computing) with the minfi, version 1.40 framework.^44^ Samples and probes returning a mean detection *P* ≥ 0.01 were omitted. ENmix, version 1.30.03 (Bioconductor) was used to normalize microarray data using the out-of-bounds method, and dye bias was corrected using RELIC.^45^ Sex chromosomes were removed, and single-nucleotide variants and cross-reactive probes were eliminated using maxprobes, version 0.02, an open-source code in R.^46^ Probe-associated bias was controlled and monocyte enrichment was corroborated by comparing DNA methylation data of each sample with known cell-sorted, monocyte-specific DNA methylation states^47^ as previously described.^48,49^ DunedinPACE scores were calculated from β matrices using the DunedinPACE package, version 0.99.^50^

### Statistical Analysis

Means were compared between groups using analysis of variance, and percentages were compared using χ^2^ tests. To account for multiple comparisons when contrasting the differences in means by sociobehavioral variables across race and ethnicity groups, adjusted *P* values were calculated by applying the Bonferroni method (2-sided *P* < .10 was considered significant). Linear regression models of DunedinPACE scores (where a score of 1.0 means equivalent biological and chronological aging) were used to examine associations with age (linear and quadratic terms), race and ethnicity, sex (female, male), and the sociobehavioral variables of NSES (low, high), BMI, educational level, diet via HEI, and PA as total hours of combined moderate and vigorous physical activities per week. All independent variables were entered continuously unless otherwise specified. Initially, a main effect model was fit. Next, the interactions between race and ethnicity and each of the sociobehavorial variables were evaluated in separate models. Models with significant interactions are presented. Significance was assessed by the Wald statistic. Covariate-adjusted means were computed by subgroup at the mean vector of other independent variables. A sensitivity analysis was performed in which educational level was parameterized categorically as less than college, college degree, and advanced degree. The intraclass correlation within census tracts for the NSES variable was not accounted for as there were too few individuals per tract for covariance estimation.

## Results

### Sample Characteristics and Associations With DunedinPACE Estimates of Biological Aging

Blood samples were collected from 650 healthy Hawaii residents from the MEC study (250 self-reported as Native Hawaiian, 200 as Japanese American, and 200 as White). Of the 650 samples, only 376 (57.8%) were suitable for data analysis, mostly due to missing or incomplete participant data on variables of interest in this study, poor cell recovery, and insufficient DNA quality or quantity (eFigure 2 in Supplement 1). Of the 376 MEC study participants included in this analysis, 113 (30.1%) self-reported as Japanese American, 144 (38.3%) as Native Hawaiian, and 119 (31.6%) as White; 189 (50.3%) were female and 187 (49.7%) were male, with no significant differences in this proportion by race and ethnicity (Table). Most participants (62.0%) lived on Oahu, with the rest on Hawaii Island (16.0%), Kauai (13.5%), and Maui (8.5%), the map of which (eFigure 1 in Supplement 1) was generated using statewide 2010 census data. Participants’ ages ranged from 45 to 76 years, with an overall mean (SE) age of 57.81 (0.38) years; age significantly varied between race and ethnicity categories. Native Hawaiian participants exhibited the youngest mean (SE) age at 55.60 (0.54) years followed by White participants at 58.22 (0.64) years and Japanese American participants at 60.19 (0.78) years. The mean (SE) DunedinPACE score for the overall population was 1.27 (0.01). Despite Native Hawaiian participants having the lowest chronological mean age in the study, the mean (SE) DunedinPACE score among these participants was significantly higher at 1.31 (0.01) than among White participants at 1.22 (0.01) and Japanese American participants at 1.25 (0.01), indicating substantial accelerated biological aging in Native Hawaiian participants. The DunedinPACE classification also differed by race and ethnicity; 161 of the overall participants (42.8%) exhibited fast DunedinPACE, defined as a DunedinPACE score of 1.29 or higher, as in other studies.^15^ The Native Hawaiian group had the highest proportion with fast DunedinPACE at 81 participants (56.3%) followed by the Japanese American group at 42 (37.2%) and the White group at 38 (31.9%). Overall, DunedinPACE and chronological age were not correlated; yet, similarly to a previous study of White adults,^15^ among Japanese American participants, a strong positive correlation was found between DunedinPACE and chronological age (eFigure 3 in Supplement 1). The mean (SE) DunedinPACE score was significantly higher among females compared with males overall (1.28 [0.01] vs 1.25 [0.01]; *P* = .005), with a significant difference between females and males observed among Native Hawaiian and Japanese American participants but not among White participants (eFigure 3 in Supplement 1).

**Table.** Summary Statistics of Study Participant Data

Variable	Participants^a^				
	Overall (N = 376)	Race and ethnicity			*P* value^b^
		Japanese American (n = 113)	Native Hawaiian (n = 144)	White (n = 119)	
Sex					
Female	189 (50.3)	55 (48.7)	72 (50.0)	62 (52.1)	.87
Male	187 (49.7)	58 (51.3)	72 (50.0)	57 (47.9)	
Age, mean (SE) [range], y	57.81 (0.38) [45.00-76.00]	60.19 (0.78) [45.00-76.00]	55.60 (0.54) [45.00-73.00]	58.22 (0.64) [45.00-74.00]	<.001
DunedinPACE score					
Mean (SE) [range]^c^	1.27 (0.01) [0.83-1.68]	1.25 (0.01) [0.99-1.54]	1.31 (0.01) [1.08-1.68]	1.22 (0.01) [0.83-1.51]	<.001
Category					
Slow	215 (57.2)	71 (62.8)	63 (43.8)	81 (68.1)	<.001
Fast	161 (42.8)	42 (37.2)	81 (56.3)	38 (31.9)	
BMI					
Mean (SE) [range]	26.72 (0.24) [20.35-38.64]	24.46 (0.31) [20.35-38.64]	28.54 (0.39) [20.35-38.64]	26.68 (0.45) [20.35-38.64]	<.001
Category					
Normal weight	154 (41.0)	67 (59.3)	37 (25.7)	50 (42.0)	<.001
Overweight	144 (38.3)	39 (34.5)	62 (43.1)	43 (36.1)	
Obese	78 (20.7)	7 (6.2)	45 (31.3)	26 (21.8)	
NSES, 1990 census, quintile					
1	21 (5.6)	4 (3.5)	11 (7.6)	6 (5.0)	.03
2	42 (11.2)	12 (10.6)	24 (16.7)	6 (5.0)	
3	89 (23.7)	25 (22.1)	38 (26.4)	26 (21.8)	
4	83 (22.1)	24 (21.2)	31 (21.5)	28 (23.5)	
5	141 (37.5)	48 (42.5)	40 (27.8)	53 (44.5)	
Educational level					
Years completed, mean (SE) [range]	14.64 (0.13) [7.00-18.00]	14.50 (0.25) [7.00-18.00]	14.07 (0.19) [7.00-18.00]	15.47 (0.22) [7.00-18.00]	<.001
Highest level completed					
Grades 6-8	7 (1.9)	4 (3.5)	2 (1.4)	1 (0.8)	<.001
Grades 9-10	5 (1.3)	1 (0.9)	2 (1.4)	2 (1.7)	
Grades 11-12	99 (26.3)	29 (25.7)	51 (35.4)	19 (16.0)	
Vocational school	33 (8.8)	19 (16.8)	10 (6.9)	4 (3.4)	
Some college	63 (16.8)	12 (10.6)	30 (20.8)	21 (17.6)	
Graduated college	84 (22.3)	23 (20.4)	31 (21.5)	30 (25.2)	
Graduate or professional school	85 (22.6)	25 (22.1)	18 (12.5)	42 (35.3)	
HEI score, mean (SE) [range]^d^	67.11 (0.49) [39.44-91.00]	67.93 (0.89) [43.41-91.00]	65.95 (0.73) [44.62-86.95]	67.75 (0.93) [39.44-89.22]	.17
Moderate or vigorous PA, mean (SE) [range], h/wk	16.89 (0.18) [8.36-23.93]	16.59 (0.35) [8.36-23.93]	17.05 (0.31) [8.96-23.43]	16.97 (0.30) [9.71-23.82]	.56
Missing PA data, No.	6	1	4	1	NA

Abbreviations: BMI, body mass index (calculated as weight in kilograms divided by height in meters squared); DunedinPACE, Dunedin Pace of Aging Calculated From the Epigenome; HEI, Healthy Eating Index; NA, not applicable; NSES, neighborhood socioeconomic status; PA, physical activity.

^a^ Data are presented as number (percentage) of participants unless otherwise indicated.

^b^ *P* values were from a Pearson χ^2^ test of independence (used for sex, DunedinPACE category, BMI category, and 1990 US census NSES) and an analysis of variance (used for chronological age, DunedinPACE, BMI, educational level, HEI score, and the number of hours of moderate or vigorous PA per week). The level of education was treated as a continuous variable measured from categorical values represented in the table and ranging from sixth grade to graduate or professional school level.

^c^ A score of 1.0 means equivalent biological and chronological aging.

^d^ Score range, 0 to 100, with higher scores indicating better alignment with key dietary recommendations that support health.

**Figure 1.**
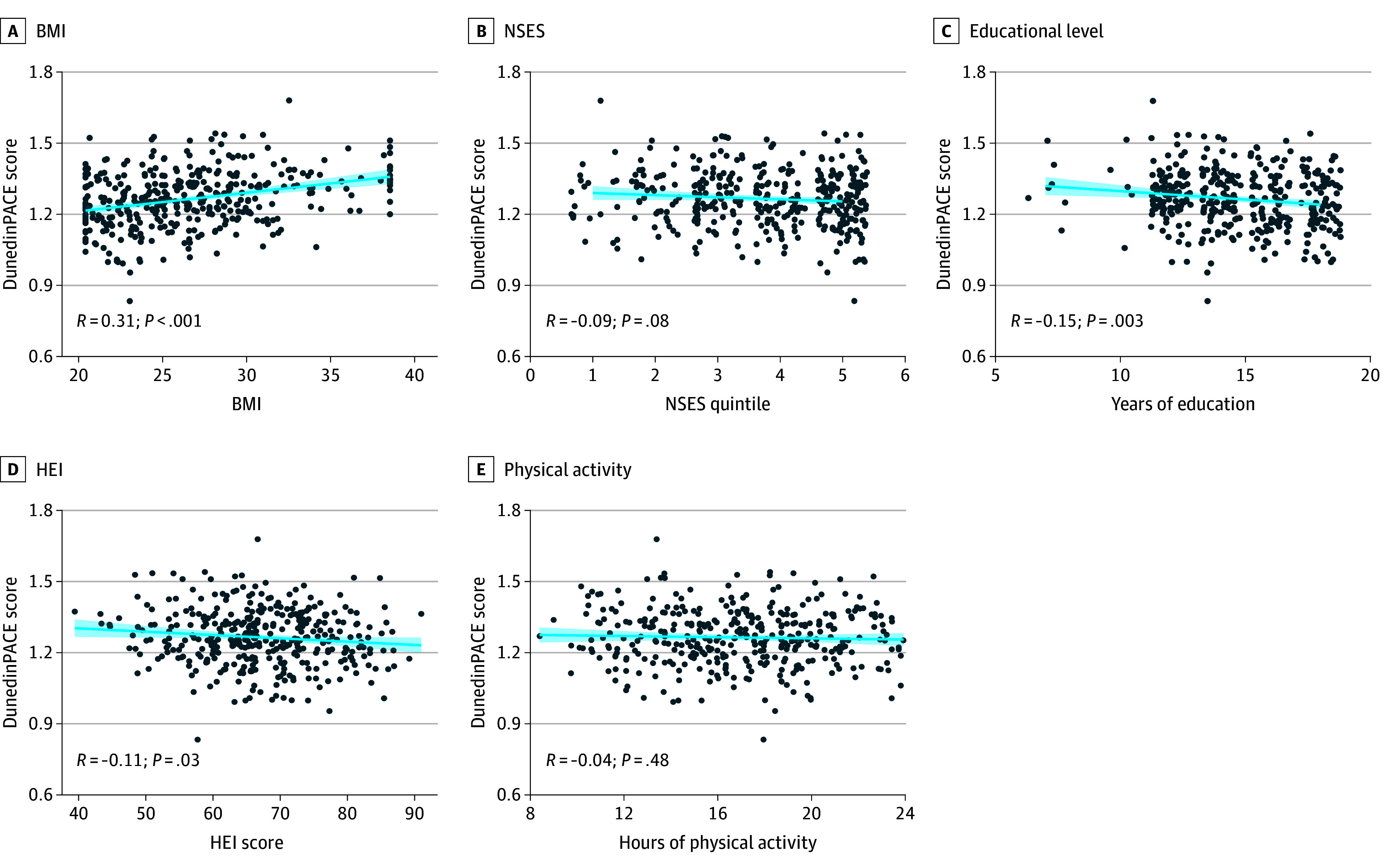
Overall Associations of Dunedin Pace of Aging Calculated From the Epigenome (DunedinPACE) Score With Sociobehavioral Factors Data points represent sample values; lines, linear trends; and shading, SE. BMI indicates body mass index (calculated as weight in kilograms divided by height in meters squared); HEI, Healthy Eating Index; NSES, neighborhood socioeconomic status.

**Figure 2.**
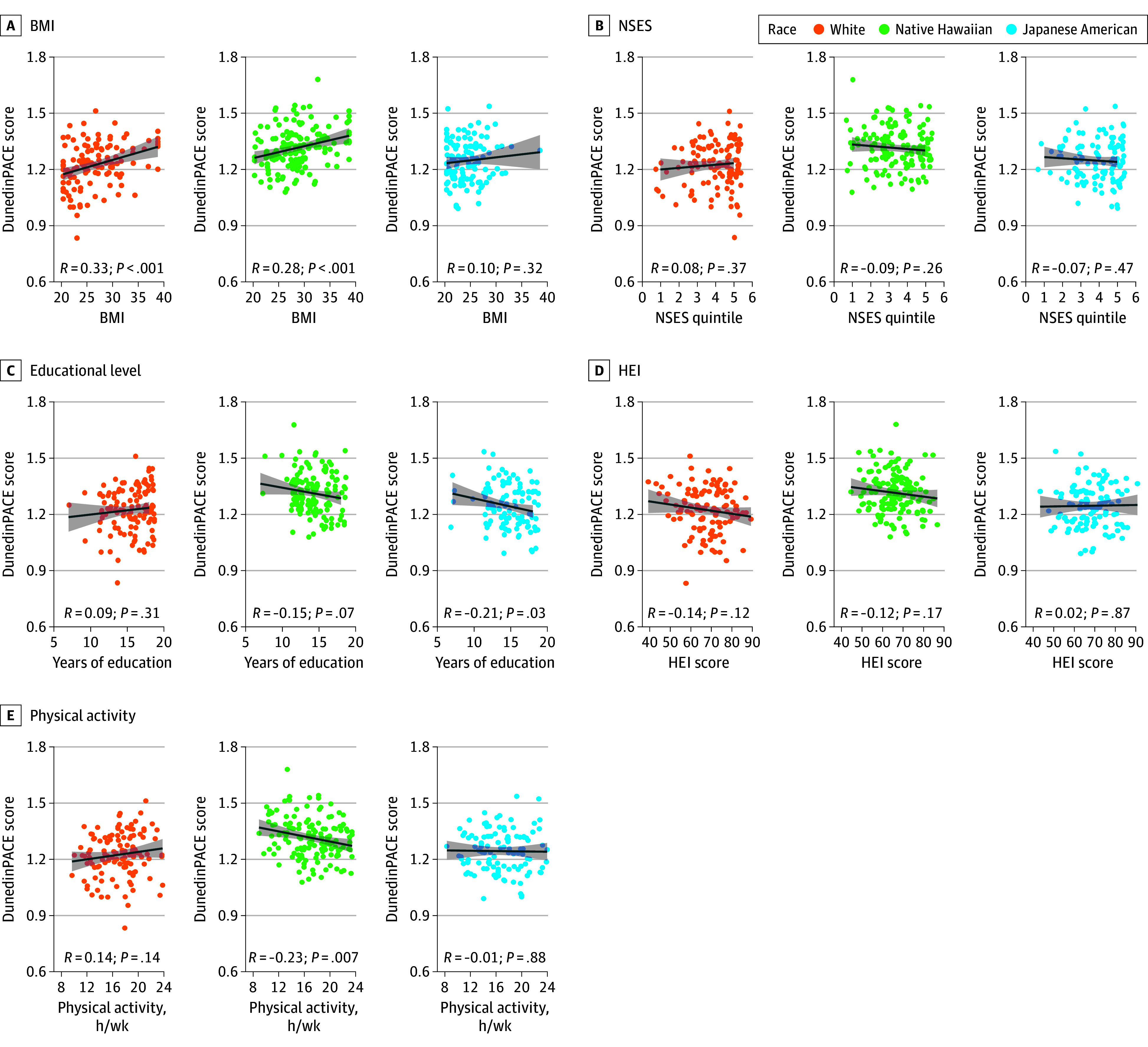
Associations Between Dunedin Pace of Aging Calculated From the Epigenome (DunedinPACE) Score and Sociobehavioral Factors by Race and Ethnicity Data points represent sample values; lines, linear trends; and shading, SE. BMI indicates body mass index (calculated as weight in kilograms divided by height in meters squared); HEI, Healthy Eating Index; NSES, neighborhood socioeconomic status.

The mean DunedinPACE scores were compared by subgroup, overall, and by race and ethnicity with Bonferroni adjustment of *P* values. Body mass index significantly varied by race and ethnicity. Native Hawaiian participants had the highest mean (SE) BMI at 28.54 (0.39) followed by White participants at 26.68 (0.45) and Japanese American participants at 24.46 (0.31). Correspondingly, the distribution of participants by BMI status significantly varied by race and ethnicity. Notably, 67 Japanese American participants (59.3%) had normal weight and 7 (6.2%) had obesity compared with 37 (25.7%) and 45 (31.3%) of Native Hawaiian participants, respectively. Overall, mean (SE) DunedinPACE scores were successively higher across BMI categories, with participants in the group with normal weight exhibiting the lowest score at 1.23 (0.01) compared with those in the groups with overweight (1.28 [0.01]; adjusted *P* = .001) and obesity (1.31 [0.01]; *P* < .001). This association was observed in White and Native Hawaiian participants but not in Japanese American participants (eFigure 4 in Supplement 1). Additionally, DunedinPACE scores were positively correlated with BMI overall (*R* = 0.31; *P* < .001), with the strongest correlations among White (*R* = 0.33; *P* < .001) and Native Hawaiian (*R* = 0.28; *P* < .001) participants (Figure 1A and Figure 2A).

Based on Hawaii NSES parameters of the MEC study established previously,^18^ the distribution of participants living in low and high NSES areas differed by race and ethnicity. Native Hawaiian participants had the lowest proportion living in high NSES areas at 71 (49.3%) compared with 81 (68.0%) for White participants and 72 (63.7%) for Japanese American participants. Overall, individuals living in low NSES areas exhibited significantly higher mean (SE) DunedinPACE scores compared with those in high NSES areas (1.28 [0.01] vs 1.25 [0.01]; *P* = .03); yet, this difference was not statistically significant in each race and ethnicity category (eFigure 4 in Supplement 1). Also, NSES and DunedinPACE scores exhibited a weak negative correlation overall (*R* = −0.09; *P* = .08), and no correlation was found among the Japanese American, Native Hawaiian, or White groups separately. However, consistent with their opposite correlations with DunedinPACE scores, BMI and NSES exhibited a significant negative correlation overall (*R* = −0.14; *P* = .008) (Figures 1B and 2B).

Educational attainment varied by race and ethnicity. Native Hawaiian participants exhibited a lower mean (SE) number of years of education at 14.07 (0.19) compared with White participants at 15.47 (0.22) (adjusted *P* < .001). Among participants overall, those with a low level of education (less than 12th grade) exhibited a significantly higher mean (SE) DunedinPACE score than those with a higher level (more than 12th grade) of education (1.29 [0.01] vs 1.25 [0.01]; *P* = .005); yet, this difference was statistically significant only among Japanese American participants (eFigure 4 in Supplement 1). Additionally, the level of education and the DunedinPACE score among participants overall were significantly negatively correlated (*R* = −0.15; *P* = .003); there was a weak negative correlation among Native Hawaiian participants (*R* = −0.15; *P* = .07) and a strong negative correlation among Japanese American participants (*R* = −0.21; *P* = .03), but a correlation was not observed among White participants (*R* = 0.09; *P* = .31) (Figures 1C and 2C).

Diet quality measured by HEI exhibited little variability by racial and ethnic group. When stratifying participants into groups with high and low HEI diet quality, pairwise comparisons showed no difference in mean (SE) HEI between Native Hawaiian participants at 65.95 (0.73) and Japanese American participants at 67.93 (0.93) (*P* = .10). No differences were observed in DunedinPACE scores between HEI groups among participants overall or by race and ethnicity (eFigure 4 in Supplement 1). However, there was a weak yet statistically significant negative correlation between HEI and DunedinPACE among participants overall (*R* = −0.11; *P* = .03); no correlations were observed among all 3 racial and ethnic groups (Figures 1D and 2D).

Overall, there were no significant differences in the level of moderate or vigorous PA between racial and ethnic groups, yet there were some associations with DunedinPACE (eFigure 4 in Supplement 1). Although the level of PA did not correlate with DunedinPACE among participants overall, Native Hawaiian participants exhibited a robust negative correlation between these variables (*R* = −0.23; *P* = .007) that was not observed in the other racial and ethnic groups (Figures 1E and 2E).

### Interactions Between Neighborhood- and Individual-Level Social Factors and DunedinPACE

**Figure 3.**
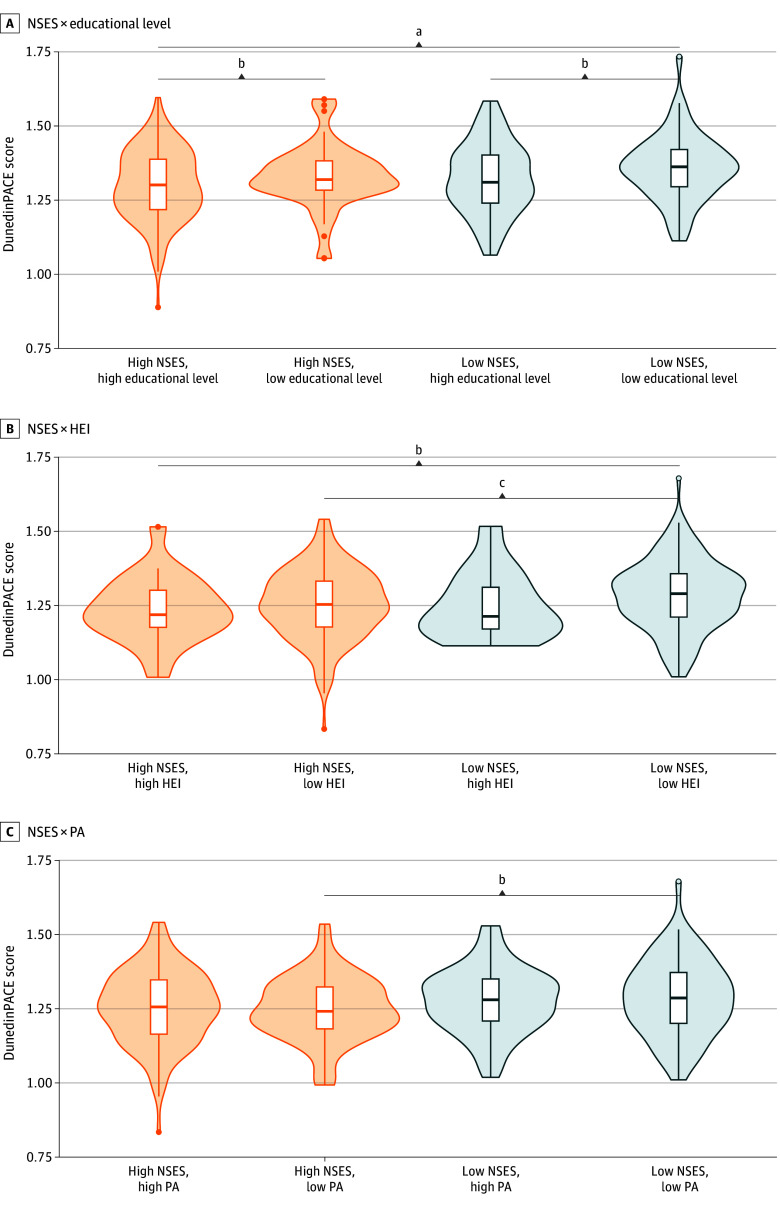
Distribution of Dunedin Pace of Aging Calculated From the Epigenome (DunedinPACE) Scores by Neighborhood Socioeconomic Status (NSES), Stratified by Educational Level, Healthy Eating Index (HEI), and Moderate or Vigorous Physical Activity (PA) in the Overall Cohort Results were from a *t* test of the differences in DunedinPACE scores. The horizontal bar inside the boxes indicates the median and the lower and upper ends of the boxes, the first and third quartiles. Shading indicates sample density. Orange indicates high SES data, and blue indicates low SES data. ^a^*P* < .001. ^b^*P* < .10. ^c^*P* < .05.

The overall associations of neighborhood- and individual-level SDOH factors and behaviors with DunedinPACE scores, coupled with differences in these associations among the 3 racial and ethnic groups in this study, indicated potential complex interactions. Such interactions were evaluated by comparing DunedinPACE scores between NSES categories further stratified by educational level, HEI, or PA among participants overall and by racial and ethnic group (unadjusted for age, sex, and BMI). Individuals with a low vs high educational level tended to show higher mean (SE) DunedinPACE scores regardless of living in areas of high NSES (low educational level: 1.28 [0.01]; high educational level: 1.25 [0.01]; *P* = .08) or low NSES (low educational level: 1.30 [0.01]; high educational level: 1.27 [0.01]; *P* = .07); those with a low educational level living in low NSES areas showed significantly higher mean (SE) DunedinPACE scores than those with a high educational level living in high NSES areas (1.30 [0.01] vs 1.25 [0.01]; *P* = .001). Notably, those with a low educational level living in low NSES areas had higher mean (SE) DunedinPACE scores than those living in low NSES areas but with a high educational level (1.30 [0.01] vs 1.27 [0.01]; *P* = .07) (Figure 3A).

Similarly, individuals with low HEI scores who also lived in areas of low NSES had higher mean (SE) DunedinPACE scores than those with high HEI scores who lived in areas with high NSES (1.28 [0.01] vs 1.24 [0.01]; *P* = .07); yet, individuals with low HEI scores who lived in areas of low NSES exhibited significantly higher mean (SE) DunedinPACE scores than those with low HEI scores who lived in areas of high NSES (1.28 [0.01] vs 1.26 [0.01]; *P* = .03) (Figure 3B). In addition, individuals living in areas of low NSES who reported low PA exhibited higher mean (SE) DunedinPACE scores compared with those living in areas of high NSES who also reported low PA (1.29 [0.01] vs 1.25 [0.01]; *P* = .05) (Figure 3C). No other notable difference in the variability of DunedinPACE scores between individuals stratified by PA and NSES was observed. Altogether, these results suggest that educational level, HEI, and PA may act as protective individual-level sociobehavioral factors that mitigate the association of low NSES with DunedinPACE, with potential differences by race and ethnicity. To test this hypothesis, a multiple linear regression analysis with interaction variables was applied.

### Estimating the Association of Social Factors With DunedinPACE

**Figure 4.**
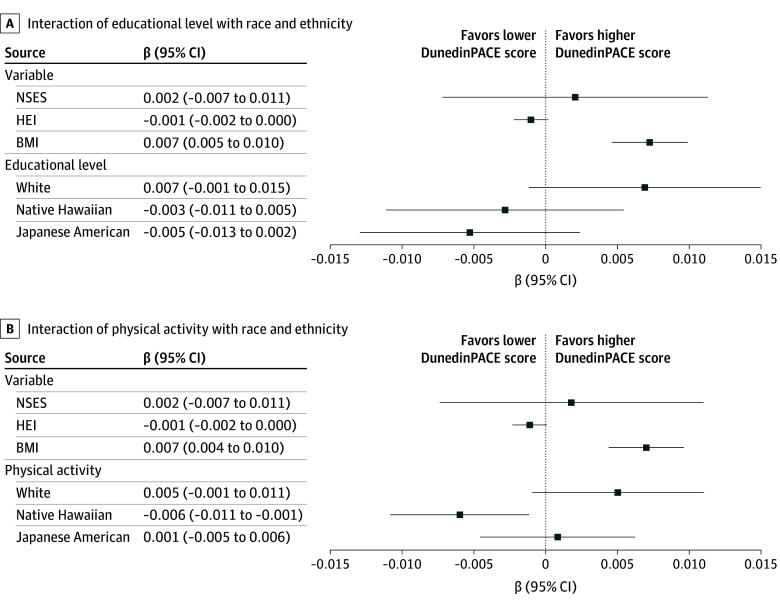
Linear Regression Analyses of Dunedin Pace of Aging Calculated From the Epigenome (DunedinPACE) Scores and Sociobehavioral Factors Models controlled for age, sex, and sociobehavioral factors. BMI indicates body mass index; HEI, Healthy Eating Index; NSES, neighborhood socioeconomic status.

Several models to consider multiple interactions between NSES, HEI, educational level, and PA overall and by race and ethnicity were performed (controlled for age, sex, and BMI); only the significant results are detailed in the eTable in Supplement 1. For the main model with the interaction between educational level and race and ethnicity, there was no association between NSES and DunedinPACE scores, whereas higher HEI scores were associated with lower DunedinPACE scores (β, −0.001; 95% CI, −0.002 to −0.001; *P* = .099) (Figure 4A). Although educational level among White participants was associated with DunedinPACE scores (β, 0.007; 95% CI, 0.001-0.015; *P* = .09), those with higher education among Japanese American and Native Hawaiian participants exhibited lower DunedinPACE scores (Japanese American: β, 0.005; 95% CI, −0.013 to 0.002; *P* = .03; Native Hawaiian: β, −0.003; 95% CI, −0.011 to 0.005; *P* = .08) (Figure 4A). The covariate-adjusted DunedinPACE score was 1.26 (0.86) for Japanese American, 1.32 (0.80) for Native Hawaiian, and 1.20 (0.82) for White participants at 12 years of education and 1.24 (0.86) for Japanese American, 1.31 (0.80) for Native Hawaiian, and 1.23 (0.82) for White participants at 16 years of education.

The level of education, treated as a categorical variable, was also used in a sensitivity model. On average, those with advanced degrees among Japanese American participants had significantly lower DunedinPACE scores than those with college degrees (β, −0.072; 95% CI, −0.142 to −0.001; *P* = .05) (eFigure 5 in Supplement 1). For the main effect model with the interaction between PA and race and ethnicity, there were significant differences in DunedinPACE scores. Like the prior model, NSES showed no association with DunedinPACE scores, whereas a higher HEI remained associated with lower DunedinPACE scores (β, −0.001; 95% CI, −0.002 to −0.001; *P* = .07) (Figure 4B). Physical activity was associated with DunedinPACE scores among Native Hawaiian participants (β, −0.006; 95% CI, −0.011 to −0.001; *P* = .005), but no association between PA and DunedinPACE was observed among Japanese American and White participants (Figure 4B). The mean adjusted DunedinPACE score was 1.24 (0.84) for Japanese American, 1.34 (0.78) for Native Hawaiian, and 1.20 (0.81) for White participants at 13 h/wk of moderate or vigorous PA and 1.25 (0.84) for Japanese American, 1.30 (0.78) for Native Hawaiian, and 1.23 (0.81) for White participants at 19 h/wk.

## Discussion

In this sample of MEC study participants, the mean DunedinPACE score was 1.27, indicating a 27% faster aging rate than that of the original Dunedin study,^15^ where a score of 1.0 means equivalent biological and chronological aging. This aging rate varied by sex, with females and males aging 28% and 25% faster, respectively, than expected. Like the original study^15^ and a recent one to examine DunedinPACE in African American adults,^33^ we found no differences in DunedinPACE scores between female and male White participants. In contrast, previous research using older epigenetic clocks noted sex differences in aging rates among Hispanic and White adults.^51,52^ Similarly, we observed that females had higher DunedinPACE scores than males among Japanese American and Native Hawaiian participants, which may reflect the higher degree of perceived stress reported among females than males living in Hawaii.^53^ To our knowledge, this is the first demonstration of sex differences in DunedinPACE estimates.

Apart from sex, we observed significant differences in biological aging rates across race and ethnicity. White participants aged 22% faster than expected, followed by Japanese American (25% faster) and Native Hawaiian (31% faster) participants, with the latter showing a considerably higher rate, attributable to the larger proportion (56.3%) of individuals having a faster DunedinPACE than the other racial and ethnic groups. Notably, the only other study to date to describe ethnic differences in DunedinPACE reported that African American adults had 7% higher DunedinPACE scores than White adults, which was attributed to poverty.^33^ We observed that Native Hawaiian participants, many of whom live in areas with low NSES, had the highest proportion (31.3%) of individuals with obesity among the 3 groups, consistent with their known health disparities.^24,25,26^ These data indicate the relevance of DunedinPACE to health among the study participants.

The strong positive correlation between DunedinPACE and BMI coupled with the negative correlation between DunedinPACE and NSES suggests that DunedinPACE may be an indicator of obesity potentially sensitive to SDOHs and behaviors.^54^ We observed that Native Hawaiian participants may be aging faster biologically than Japanese American or White participants, which may be associated with obesity and is likely reflected in the socioeconomic inequities they experience. However, results of our regression models indicated that higher educational attainment may mitigate high rates of biological aging in Japanese American and Native Hawaiian individuals. A healthy diet was also associated with DunedinPACE across participants overall. Additionally, among Native Hawaiian participants, higher physical activity was negatively correlated with DunedinPACE, indicating a possible protective association, while there was no association among Japanese American participants and an unexpected positive correlation among White participants, which may indicate that physical activity is associated with accelerated aging and may support the unexplained elevated cardiovascular risk of excessive exercise among White adults.^55^ We recognize that the association between physical activity and aging may be complex and variable according to the type of epigenetic clock used.^54,56,57,58,59,60,61^

Our findings showed a positive association of diet, educational level, and physical activity with reduced DunedinPACE irrespective of NSES in some racial and ethnic groups, suggesting that protective sociobehavioral factors may mitigate the association of NSES with biological aging. To our knowledge, this is the first study on epigenetic clocks, specifically DunedinPACE, in an underrepresented population of adults who self-identified as Japanese American or Native Hawaiian. Our results have implications for health disparities research and reinforce that race and ethnicity may account for social experiences and behaviors that impact epigenetic regulation of aging. Consequently, DunedinPACE may be considered a social epigenomic biomarker with generalizable relevance to racially and ethnically diverse populations, warranting further investigation.

### Limitations

This study has limitations. The elevated DunedinPACE scores in our study compared with the original Dunedin study^15^ could stem from several factors: the older mean age of the participants in our study, our use of monocytes (vs peripheral blood mononuclear cells) for DNA methylation analysis, and unmeasured socioenvironmental conditions. Additionally, the racial and ethnic diversity in our study, including Japanese American and Native Hawaiian populations underrepresented in prior biomedical research, may have introduced variation in DNA methylation and unrecognized genetic differences that could affect the validity of DunedinPACE and other epigenetic clocks, as they were derived predominantly from populations of European ancestry. Also, a larger sample size for each racial and ethnic group would have increased our statistical power in subgroup analyses, would have allowed for accounting for NSES intraclass correlations, and would be needed to confirm our findings. To enhance precision and generalizability, future clocks should be trained on and calibrated with DNA methylation data from these underrepresented populations.

## Conclusions

This multiethnic cohort study of 376 adults revealed significant racial and ethnic diversity in the DunedinPACE estimates of biological aging that were associated with specific SDOHs. Using DunedinPACE and possibly other epigenetic clocks, future studies can potentially improve disease risk assessment and identify sociobehavioral factors that protect against rapid biological aging, informing early detection and prevention strategies, respectively.

## Notes

Supplement 1. **eFigure 1.** Cohort Participants Residing in Areas of Diverse NSES **eFigure 2.** Flow Chart of Inclusion and Exclusion Criteria of Samples From Cohort Participants **eFigure 3.** Associations Between Age, DunedinPACE, Race and Ethnicity, and Sex **eFigure 4.** Distribution of DunedinPACE by Sociobehavioral Factors **eFigure 5.** Linear Regression Analyses of DunedinPACE and Sociobehavioral Factors With Educational Level as Categories **eTable.** Data of Linear Regression Analyses of DunedinPACE and Sociobehavioral Factors

Supplement 2.
Data Sharing Statement
