# Supplementary material for: Socioeconomic Status, Lifestyle, and DNA Methylation Age Among Racially and Ethnically Diverse Adults: NIMHD Social Epigenomics Program
Source: JAMA Netw Open. 2024 Jul 29;7(7):e2421889. doi: 10.1001/jamanetworkopen.2024.21889 (PMC11287425; doi:10.1001/jamanetworkopen.2024.21889)
Supplement: Supplement 1. — eFigure 1. Cohort Participants Residing in Areas of Diverse NSES eFigure 2. Flow Chart of Inclusion and Exclusion Criteria of Samples From Cohort Participants eFigure 3. Associations Between Age, DunedinPACE, Race and Ethnicity, and Sex eFigure 4. Distribution of DunedinPACE by Sociobehavioral Factors eFigure 5. Linear Regression Analyses of DunedinPACE and Sociobehavioral Factors With Educational Level as Categories eTable. Data of Linear Regression Analyses of DunedinPACE and Sociobehavioral Factors [file jamanetwopen-e2421889-s001.pdf]

Supplementary Online Content

Maunakea AK, Phankitnirundorn K, Peres R, et al. Socioeconomic status, lifestyle, and DNA methylation age among racially and ethnically diverse adults: NIMHD Social Epigenomics Program. *JAMA Netw Open*. 2024;7(7):e2421889. doi:10.1001/jamanetworkopen.2024.21889

eFigure 1. Cohort Participants Residing in Areas of Diverse NSES

eFigure 2. Flow Chart of Inclusion and Exclusion Criteria of Samples From Cohort Participants

eFigure 3. Associations Between Age, DunedinPACE, Race and Ethnicity, and Sex

eFigure 4. Distribution of DunedinPACE by Sociobehavioral Factors

eFigure 5. Linear Regression Analyses of DunedinPACE and Sociobehavioral Factors With Educational Level as Categories

eTable. Data of Linear Regression Analyses of DunedinPACE and Sociobehavioral Factors

This supplementary material has been provided by the authors to give readers additional information about their work.

© 2024 Maunakea AK et al. *JAMA Network Open*.

eFigure 1. Cohort Participants Reside in Areas of Diverse nSES.

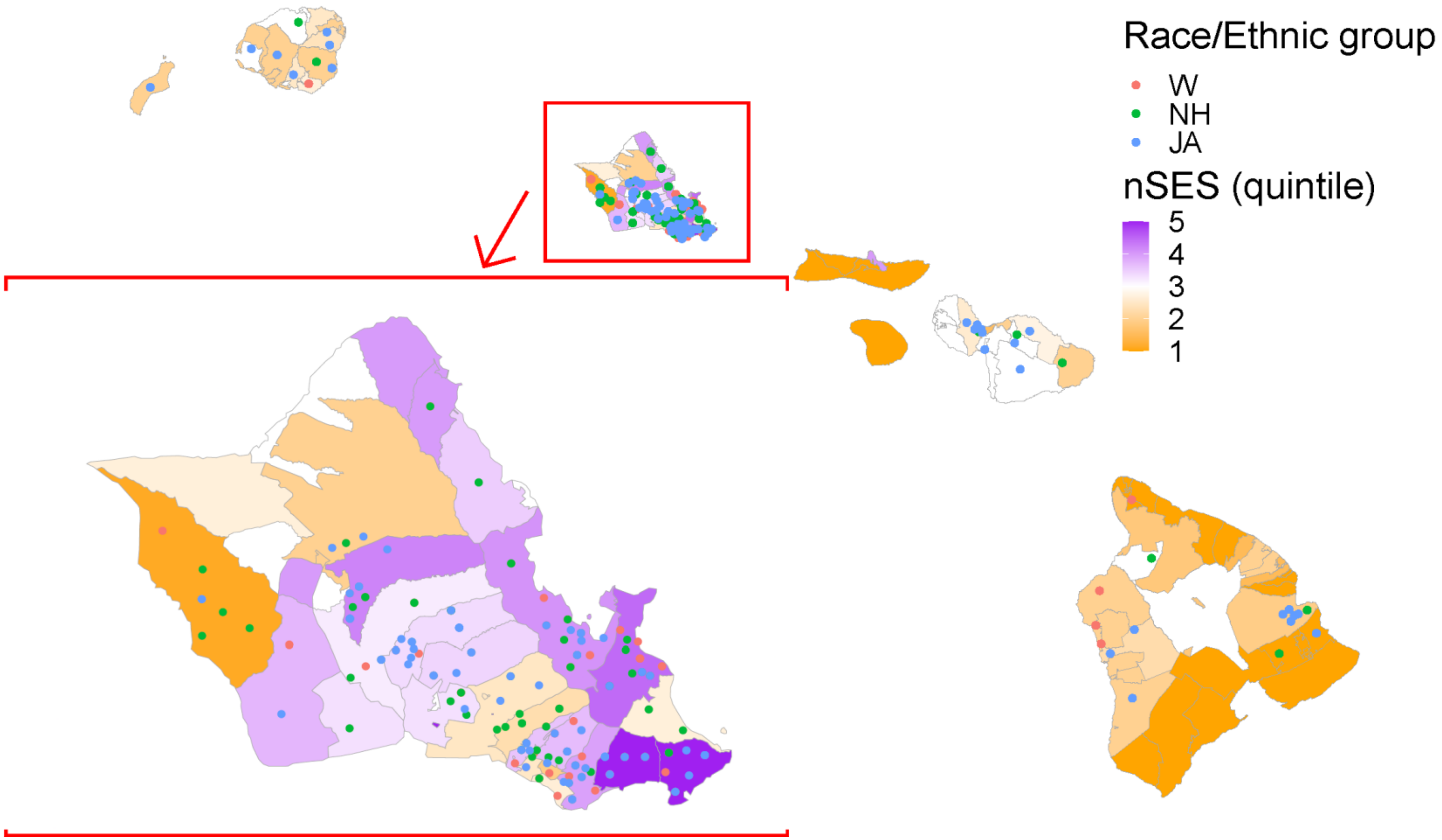

Heatmap of nSES (from 2010 U.S. Census data) across the state of Hawaii along with participants' 1990 U.S. Census tract residential locations. Dots represent individual participants, the color of which represent their self-reported primary racial/ethnic group. W: White, NH: Native Hawaiian, and JA: Japanese American participants. The 2010 U.S. Census data of nSES quintiles from Q1 to Q5 are shown in the heatmap, which strongly correlated with the 1990 data (R = 0.80, *P* < 0.001).

© 2024 Maunakea AK et al. *JAMA Network Open*.

eFigure 2. Flow Chart of Inclusion and Exclusion Criteria of Samples from Cohort Participants.

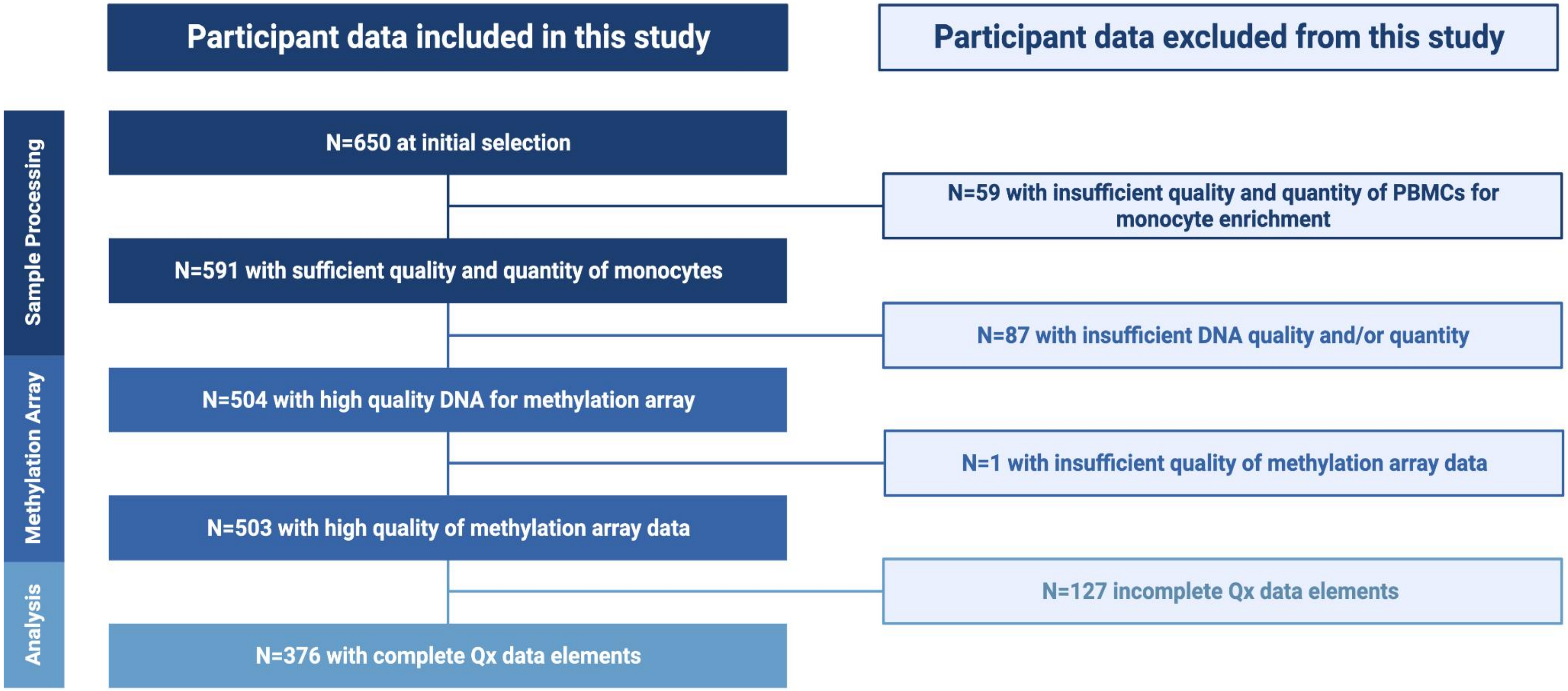

© 2024 Maunakea AK et al. *JAMA Network Open*.

eFigure 3. Relationship of Age, DunedinPACE, Race/Ethnicity and Sex.

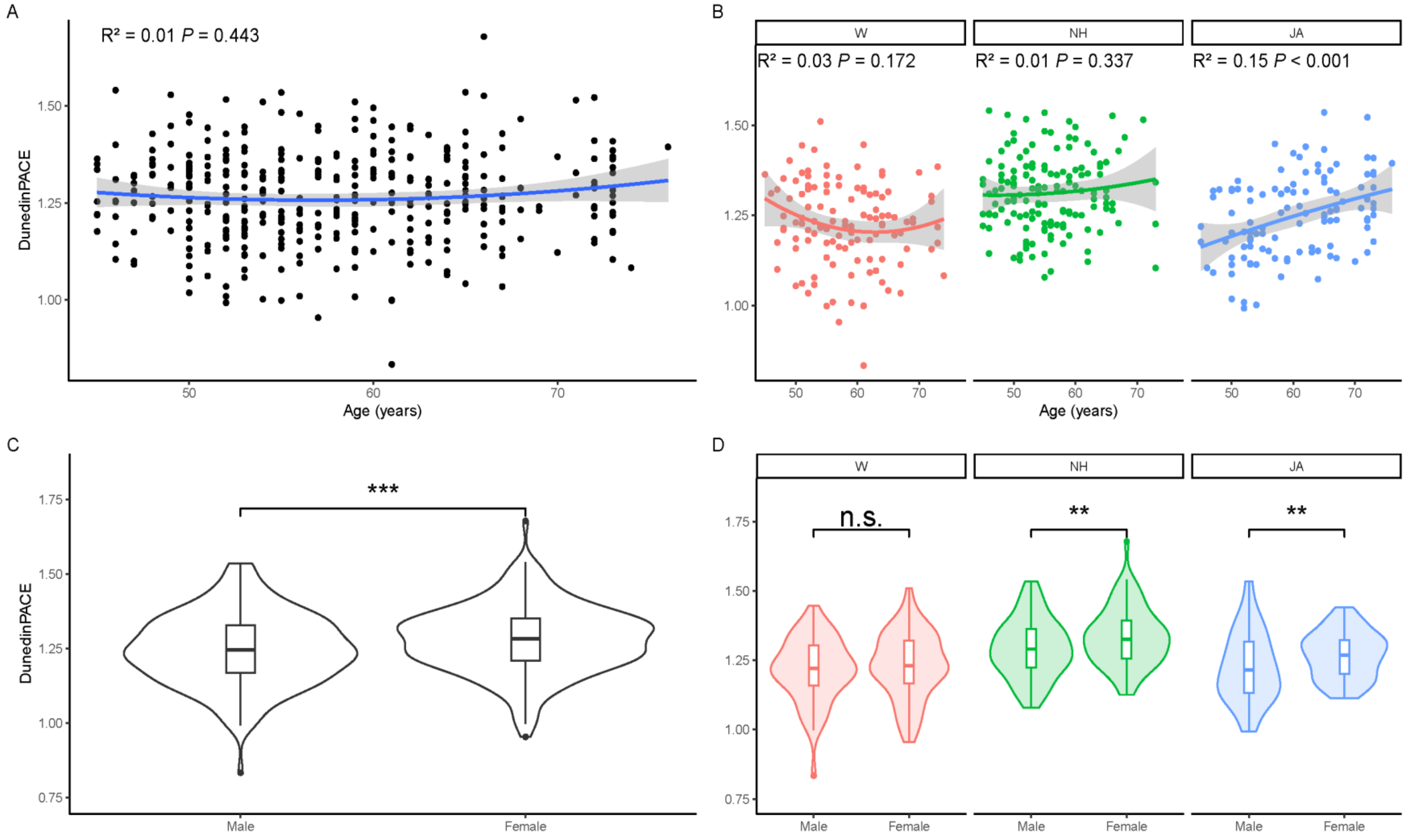

Graphs show the quadratic relationship and standard error between age and DunedinPACE in the overall cohort (**A**) and stratified by race/ethnicity (**B**). The correlation coefficients (R) and *P*-values are shown. Violin plots and t-test of the differences in DunedinPACE between sexes of participants in the overall cohort (**C**) and stratified by race/ethnicity (**D**). W: White, NH: Native Hawaiian, and JA: Japanese American participants. ***, *P*<0.001; **, *P*<0.05; *, *P*<0.10; n.s., non-significant.

© 2024 Maunakea AK et al. *JAMA Network Open*.

eFigure 4. Distribution of DunedinPACE by Sociobehavioral Factors.

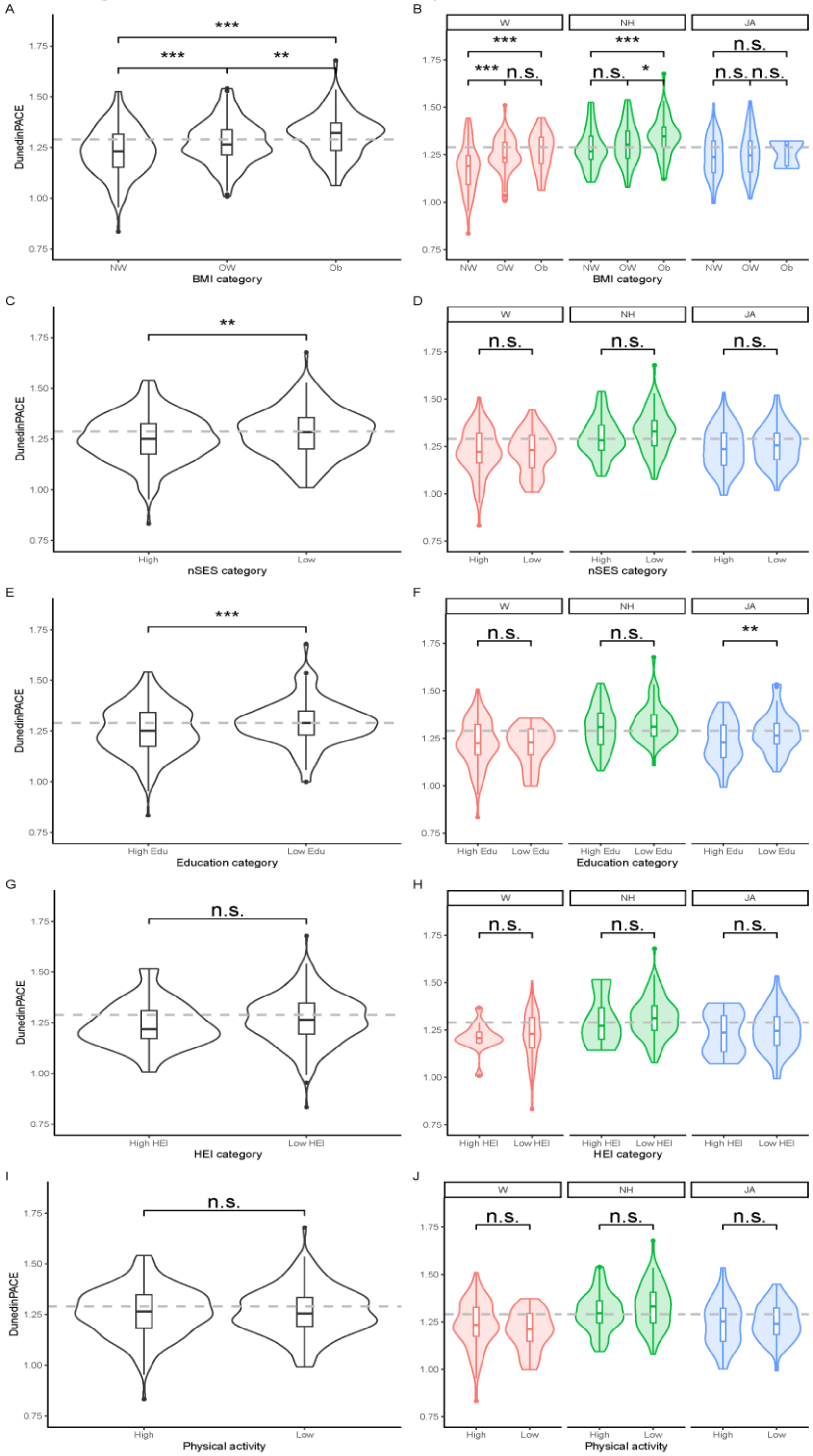

Violin plot of DunedinPACE by sociobehavioral factors (BMI, nSES, education, HEI, and PA) and a t-test of the differences in DunedinPACE between these variables in the overall cohort (**A, C, E, G,** and **I**, respectively) and stratified by race/ethnicity (**B, D, F, H,** and **J,** respectively). NW: normal weight; OW: Overweight; Ob: Obese. W: White, NH: Native Hawaiian, and JA: Japanese American participants. ***, *P*<0.001; **, *P*<0.05; *, *P*<0.10; n.s., non-significant.

© 2024 Maunakea AK et al. *JAMA Network Open*.

eFigure 5. Linear Regression Analyses of DunedinPACE and Sociobehavioral Factors with Education as Categories.

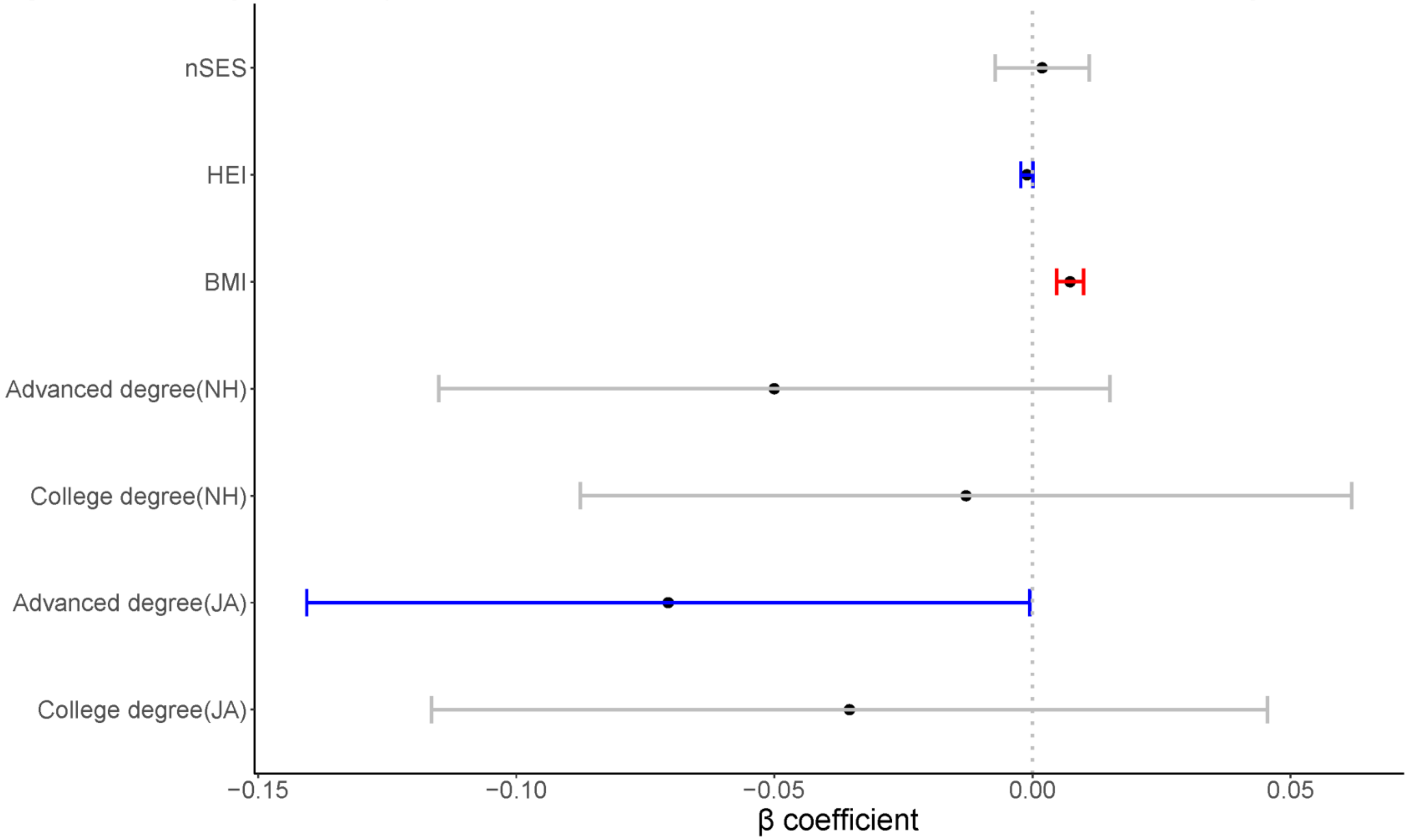

Graph of beta coefficients with 95% confidence interval from the model with interaction between education and race/ethnicity, controlling for age, sex and other sociobehavioral factors. Education was categorized as with GED, some college degree, and advanced degree where GED was used as a baseline. NH: Native Hawaiian; JA: Japanese American. ***, *P*<0.001; **, *P*<0.05; *, *P*<0.10.

© 2024 Maunakea AK et al. *JAMA Network Open*.

	DunedinPACE score (dependent variable)			
	**Model 1**	**Model 2**	**Model 3**	**Model 4**
**Age, years old**	-0.012	-0.005	-0.003	-0.009
	(0.012)	(0.011)	(0.011)	(0.011)
**Age^2, years old**	0.0001	0.0001	0.0001	0.0001
	(0.0001)	(0.0001)	(0.0001)	(0.0001)
**Sex (female)**	0.032***	0.027**	0.030***	0.027**
	(0.012)	(0.011)	(0.011)	(0.011)
**nSES, quintile**		0.002	0.002	0.002
		(0.005)	(0.005)	(0.005)
**HEI**		-0.001	-0.001*	-0.001*
		(0.001)	(0.001)	(0.001)
**BMI, Kg/m^2**		0.007***	0.007***	0.007***
		(0.001)	(0.001)	(0.001)
**Education, years**		-0.001	0.007*	-0.001
		(0.002)	(0.004)	(0.002)
**Activity, hours/week**		-0.001	-0.001	0.005*
		(0.002)	(0.002)	(0.003)
**Race (NH)**		0.085***	0.232***	0.272***
		(0.014)	(0.086)	(0.067)
**Race (JA)**		0.031**	0.215**	0.101
		(0.015)	(0.086)	(0.069)
**Education x NH**			-0.010*	
			(0.006)	
**Education x JA**			-0.012**	
			(0.006)	
**Activity x NH**				-0.011***
				(0.004)
**Activity x JA**				-0.004
				(0.004)
**Constant**	1.589***	1.150***	0.990***	1.180***
	(0.357)	(0.347)	(0.356)	(0.346)
**Observations**	376	370	370	370
**R^2**	0.024	0.229	0.240	0.247
**Adjusted R^2**	0.017	0.208	0.215	0.222
**Residual Std. Error**	0.117 (df = 372)	0.106 (df = 359)	0.105 (df = 357)	0.105 (df = 357)
**F Statistic**	3.113** (df = 3; 372)	10.668*** (df = 10; 359)	9.401*** (df = 12; 357)	9.767*** (df = 12; 357)

***, P<0.001; **, P<0.05; *, P<0.10.

eTable. Data of Linear Regression Analyses of DunedinPACE and Sociobehavioral Factors

Detailed results of linear regression analyses of DunedinPACE on sociobehavioral factors including demographic variables (age, sex, race/ethnicity), HEI, BMI, nSES, education, and PA. Data from White participants served as baseline. Model 1: null model. Model 2: main effect model. Model 3: Model 2 with the interaction between education (years) and race/ethnicity. Model 4: Model 2 with the interaction between PA and race/ethnicity. NH: Native Hawaiian; JA: Japanese American.

© 2024 Maunakea AK et al. *JAMA Network Open*.
