# Supplementary material for: Socioeconomic Status, Lifestyle, and DNA Methylation Age Among Racially and Ethnically Diverse Adults: NIMHD Social Epigenomics Program
Source: JAMA Netw Open. 2024 Jul 29;7(7):e2421889. doi: 10.1001/jamanetworkopen.2024.21889 (PMC11287425; doi:10.1001/jamanetworkopen.2024.21889)
Supplement: Supplement 2. — Data Sharing Statement [file jamanetwopen-e2421889-s002.pdf]

Data Sharing Statement

Maunakea. Socioeconomic Status, Lifestyle, and DNA Methylation Age Among Racially and Ethnically Diverse Adults. *JAMA Netw Open*. Published July 29, 2024.
doi:10.1001/jamanetworkopen.2024.21889

Data
Data available: Yes
Data types: Deidentified participant data
How to access data: All data used for this study are available de-identified upon reasonable request made to the MEC Research Committee at: https://uhcancercenter.org/for-researchers/mec-data-sharing. The datasets used in this study are available in the National Center for Biotechnology Information (NCBI) website's Gene Expression Omnibus (GEO) at https://www.ncbi.nlm.nih.gov/geo/browse/ corresponding to the following GEO Accession number: GSE270223
When available: With publication

Supporting Documents
Document types: None

Additional Information
Who can access the data: Researchers whose proposed use of the data has been approved
Types of analyses: For any purpose
Mechanisms of data availability: With a signed data access agreement
